# Heterozygote manifestations in X-chromosomal ectodermal dysplasia

**DOI:** 10.1186/1746-160X-8-S1-I11

**Published:** 2012-05-25

**Authors:** Andreas Gal

**Affiliations:** 1 Institute of Human Genetics, University Medical Center Hamburg-Eppendorf, Hamburg, Germany

## 

About 60-70 % of the heterozygotes of X-linked hypohidrotic ectodermal dysplasia (XLHED) show some clinical manifestations of the disease. Dental abnormalities are key diagnostic features and can be best evaluated at a young age. Compared to controls, carriers have a significantly higher frequency of agenesis of permanent teeth with persistence of deciduous teeth, small teeth resulting in gaps between the teeth, and peg-shaped teeth. Whole saliva flow seems to be reduced in carriers, whereas concentrations of most inorganic salivary constituents and total protein are higher than in controls. Craniofacial morphology of carriers shows some subtle deviations such as short and retrognatic maxilla, protruding lips, and a shorter total facial height compared to controls, findings that could be explained in part by hypodontia. Mild hypotrichosis and mild hypohidrosis are two further commonly seen carrier signs. No correlation has been found so far between the type of mutations, the patients’ phenotypes and disease severity. The mosaic-like distribution of normal and abnormal skin along the Blaschko lines is a typical consequence of the clonal inactivation of the X-chromosomes. While carriers with a highly skewed X-chromosome inactivation pattern may show pronounced clinical features due to the unequal expression of the two alleles (‘functional hemizygosity’), no correlation has been found between the severity of clinical symptoms and X-chromosome inactivation in leukocytes of carriers. Sporadic cases of males with XLHED and autosomal recessive HED (arHED) cannot be distinguished clinically. In contrast to heterozygotes of XLHED, who frequently show mild involvement, heterozygous parents of patients with arHED show no features of the disorder. However, because of the highly variable expression of XLHED in carriers, phenotypic assessment may result both in underdetection and false-positive diagnosis.

